# Gut microbiota depletion by chronic antibiotic treatment alters the sleep/wake architecture and sleep EEG power spectra in mice

**DOI:** 10.1038/s41598-020-76562-9

**Published:** 2020-11-11

**Authors:** Yukino Ogawa, Chika Miyoshi, Nozomu Obana, Kaho Yajima, Noriko Hotta-Hirashima, Aya Ikkyu, Satomi Kanno, Tomoyoshi Soga, Shinji Fukuda, Masashi Yanagisawa

**Affiliations:** 1 International Institute for Integrative Sleep Medicine (WPI-IIIS), University of Tsukuba, 1-1-1 Tennodai, Tsukuba, Ibaraki 305-8575 Japan; 2 Institute for Advanced Biosciences, Keio University, 246-2 Mizukami, Kakuganji, Tsuruoka, Yamagata 997-0052 Japan; 3 Food Research Institute, National Agriculture and Food Research Organization (NARO), 2-1-12 Kannondai, Tsukuba, Ibaraki 305-8642 Japan; 4 Transborder Medical Research Center, University of Tsukuba, 1-1-1 Tennodai, Tsukuba, Ibaraki 305-8575 Japan; 5 Intestinal Microbiota Project, Kanagawa Institute of Industrial Science and Technology, 3-25-13 Tonomachi, Kawasaki-ku, Kawasaki, Kanagawa 210-0821 Japan; 6 Metabologenomics, Inc., 246-2 Mizukami, Kakuganji, Tsuruoka, Yamagata 997-0052 Japan; 7 Life Science Center for Survival Dynamics, Tsukuba Advanced Research Alliance (TARA), University of Tsukuba, 1-1-1 Tennodai, Tsukuba, Ibaraki 305-8575 Japan; 8 R&D Center for Frontiers of Mirai in Policy and Technology (F-MIRAI), University of Tsukuba, 1-1-1 Tennodai, Tsukuba, Ibaraki 305-8575 Japan; 9 Department of Molecular Genetics, University of Texas Southwestern Medical Center, Dallas, TX 75390-8584 USA

**Keywords:** Sleep, Microbiome, Metabolomics

## Abstract

Dysbiosis of the gut microbiota affects physiological processes, including brain functions, by altering the intestinal metabolism. Here we examined the effects of the gut microbiota on sleep/wake regulation. C57BL/6 male mice were treated with broad-spectrum antibiotics for 4 weeks to deplete their gut microbiota. Metabolome profiling of cecal contents in antibiotic-induced microbiota-depleted (AIMD) and control mice showed significant variations in the metabolism of amino acids and vitamins related to neurotransmission, including depletion of serotonin and vitamin B6, in the AIMD mice. Sleep analysis based on electroencephalogram and electromyogram recordings revealed that AIMD mice spent significantly less time in non-rapid eye movement sleep (NREMS) during the light phase while spending more time in NREMS and rapid eye movement sleep (REMS) during the dark phase. The number of REMS episodes seen in AIMD mice increased during both light and dark phases, and this was accompanied by frequent transitions from NREMS to REMS. In addition, the theta power density during REMS was lower in AIMD mice during the light phase compared with that in the controls. Consequently, the gut microbiota is suggested to affect the sleep/wake architecture by altering the intestinal balance of neurotransmitters.

## Introduction

Sleep is a ubiquitous, indispensable brain function that maintains physiological homeostasis. Despite its importance, people living in modern society often suffer from sleep problems such as daytime sleepiness and insomnia. Sleep is strongly affected by internal and external environmental cues, including circadian rhythms and feeding. Food choice and the misalignment of feeding habits (i.e., what and when to eat) affect the size, composition, and diurnal rhythmicity of the gut microbiota^1–3^. Fluctuations in the gut microbiota cause the alteration of metabolic state because many of the metabolites found in the intestinal lumen are produced by the microbiota, which processes compounds derived from foods^4–9^. The intestinal metabolic state is closely connected to brain function through the circulatory system and vagus nerve. These interactions are known as the “brain–gut axis” or “microbiota–gut–brain axis”^9,10^. Dysbiosis of the gut microbiota leads to the impairment of brain functions such as memory formation, cognitive function, mental health, and circadian rhythmicity^9,11–13^. Accordingly, the gut microbiota may also affect the sleep/wake cycle, representing fundamental brain state transitions. However, the impact of gut microbiota on sleep regulation has not been evaluated precisely.

In this study, we analyzed the electroencephalogram (EEG)/electromyogram (EMG)-based sleep/awake architecture to examine whether the gut microbiota contributes to sleep/wake regulation. Feeding habits can simultaneously alter the composition of the gut microbiota and stimulate peripheral sensations that evoke a variety of signals that travel to the brain. To evaluate the effect of the gut microbiota on sleep while excluding feeding signals, antibiotic-induced microbiota-depleted (AIMD) mice, which were treated for 4 weeks with broad-spectrum antibiotics dissolved in drinking water, were compared with control mice drinking normal water^6,8,14^. Metabolome profiling of cecal contents was performed to compare the AIMD and control conditions and to determine which metabolites might be involved in sleep/wake regulation. Then, the wakefulness, non-rapid eye movement sleep (NREMS), and rapid eye movement sleep (REMS) states were determined in the AIMD and control mice according to the EEG/EMG definition of sleep to reveal detailed structural alterations and effects on the sleep EEG spectrum.

## Results

### Alteration of neurotransmission-related gut metabolites in the AIMD condition

The numbers of viable anaerobic bacteria in the fecal samples were 11.17 ± 5.58 × 10^4^ in the AIMD group (*n* = 13) after 4 weeks of antibiotic treatment and > 4.25 ± 1.16 × 10^9^ in the control group (*n* = 11; one sample contained too many to count). The number of intestinal microorganisms in the AIMD mice was 10^5^-fold less than that in the controls (*p* = 0.004, Welch’s *t* test). The body weight of the mice after 4 weeks of antibiotic treatment did not differ significantly between the AIMD (26.12 ± 0.27 g, *n* = 13) and control (25.41 ± 0.50 g, *n* = 12) groups (*p* = 0.228, Welch’s *t* test).

**Figure 1:**
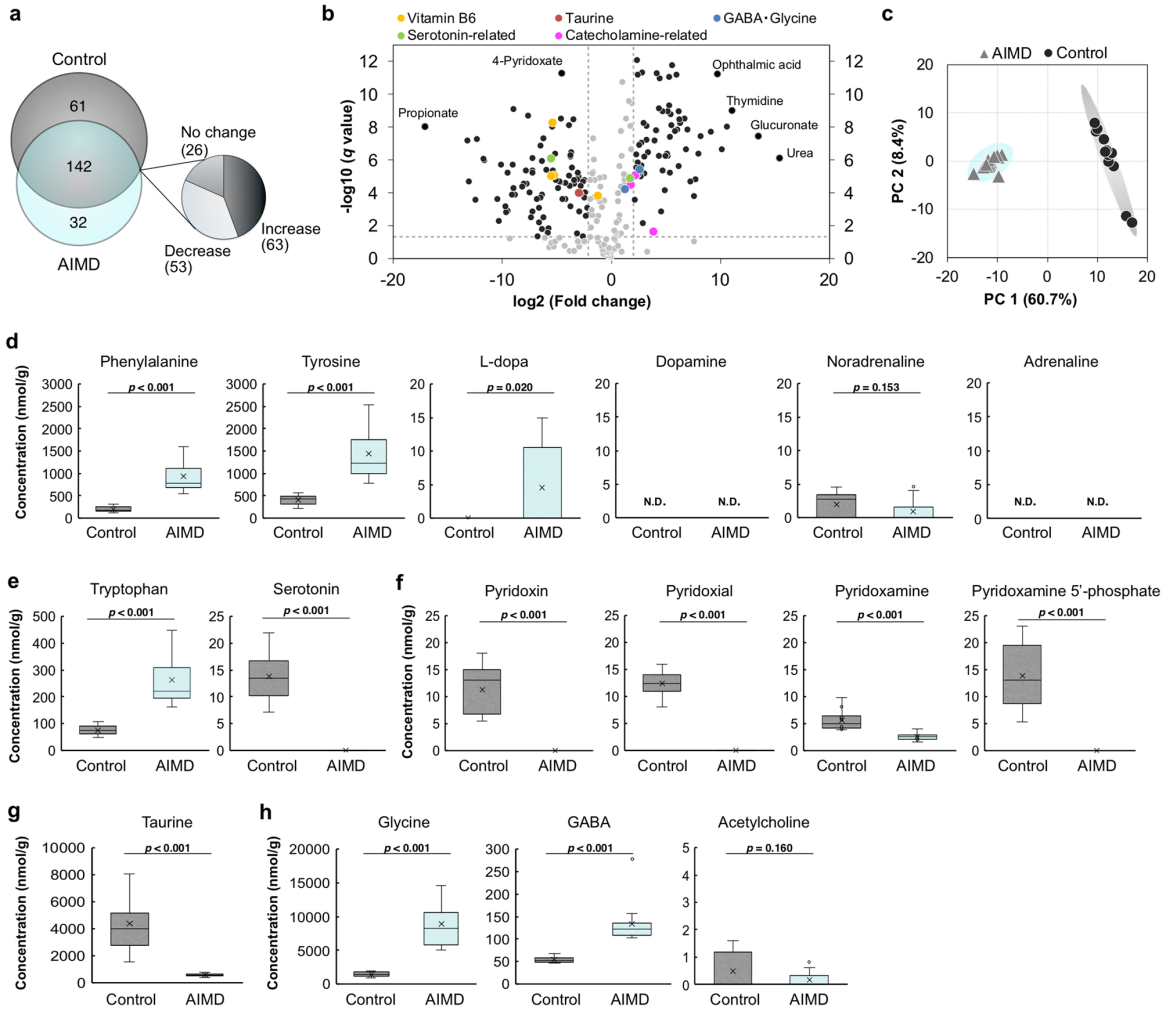
Metabolome profiling of cecal contents in the antibiotic-induced microbiota-depleted (AIMD) and control groups. (**a**) Number of metabolites in the cecal contents of the AIMD (*n* = 13) and/or control (*n* = 12) groups as detected by CE-TOFMS. (**b**) Volcano plot of metabolites that were increased and decreased in the AIMD group (*n* = 13) compared with the control group (*n* = 12). The possible mean differences between the AIMD and control groups were assessed using one-way analysis of variance (ANOVA) followed by post hoc Benjamini–Hochberg false discovery rate correction (*q* value). The *black* and *gray dots* represent the metabolites that, respectively, did or did not change significantly under the AIMD condition. The *dashed lines* indicate the threshold of fold changes on the x-axis (> 2.0) and *q* values on the y-axis (> 0.05). (**c**) Principal component analysis (PCA) of the metabolome profiles of the cecal contents. The *circles* and *triangles* represent the control (*n* = 12) and AIMD (*n* = 13) groups, respectively. (**d–h**) Concentrations of metabolites in the cecal contents, including phenylalanine, tyrosine, L-dopa, dopamine, noradrenaline, and adrenaline, in the catecholamine synthesis pathway (**d**) and tryptophan and serotonin in the serotonin synthesis pathway (**e**). The concentrations of vitamin B6, e.g., pyridoxin, pyridoxal, pyridoxamine, and pyridoxamine phosphate (**f**), taurine (**g**), glycine, GABA, and acetylcholine (**h**). The average value is indicated by the *x-ma*rk in each box plot. Values outside of a ± twofold standard deviation range are plotted as *open circles*. The possible mean difference was assessed using two-tailed Welch’s *t* test. *N.D.* not detected.

The cecal contents of the AIMD and control mice were analyzed using capillary electrophoresis-time-of-flight mass spectrometry (CE-TOFMS)^15,16^, which detected 246 metabolites in total. Significant differences in 209 of the detected metabolites were seen between the two experimental groups (Fig. 1a,b). A number of metabolites were unique to the control group (61 species, including propionate; Fig. 1b and Supplementary Table S1 online) and AIMD group (32 species, including urea, thymidine, and ophthalmic acid; Fig. 1b and Supplementary Table S2 online). Of the 142 metabolites detected in both groups, 63 compounds, including glucuronate, and 53 metabolites, including 4-pyridoxate, increased and decreased significantly in the AIMD group, respectively (Fig. 1b and Supplementary Table S3 online). Principal component analysis (PCA) of the metabolome profiles showed a clear separation between the AIMD and control groups (Fig. 1c). Ninety-one and 81 of the entire group of 246 detected metabolites showed high positive (*r* > 0.7) and negative (*r* <  − 0.7) factor loading values, indicating a strong correlation with the first principal component (PC1), respectively (Supplementary Tables S1–S3 online).

Functional annotation of the differentially expressed metabolites was carried out using the metabolome set enrichment analysis (MSEA). The findings indicated that 30 canonical pathways were significantly affected by the AIMD condition (Table 1). The major biological functions of these pathways were amino acid metabolism (50%) and carbohydrate metabolism (27%). Others included metabolism of cofactors and vitamins (7%), lipids (7%), nucleotides (7%), and translation of genetic information (3%).

**Table 1:** Metabolic pathways significantly changed in the cecal contents of antibiotic-induced microbiota-depleted (AIMD) mice.

Pathway	KEGG pathway id	Number of compounds	Impact	*p* value (FDR)
		Hits/total		
Phenylalanine, tyrosine and tryptophan biosynthesis	map00400	2/4	1.00	1.90E−06
Taurine and hypotaurine metabolism	map00430	5/8	1.00	3.57E−06
Alanine, aspartate and glutamate metabolism	map00250	13/28	0.78	3.01E−06
Glycine, serine and threonine metabolism	map00260	14/34	0.77	9.65E−11
Vitamin B6 metabolism	map00750	5/9	0.70	1.94E−16
Histidine metabolism	map00340	8/16	0.67	1.33E−04
Arginine biosynthesis	map00220	12/14	0.60	3.57E−10
Nicotinate and nicotinamide metabolism	map00760	7/15	0.60	2.00E−02
beta-Alanine metabolism	map00410	9/21	0.56	9.12E−03
Arginine and proline metabolism	map00330	13/38	0.51	4.99E−12
D-Glutamine and D-glutamate metabolism	map00471	3/6	0.50	1.61E−05
Cysteine and methionine metabolism	map00270	12/33	0.47	1.47E−07
Pyrimidine metabolism	map00240	11/39	0.39	1.42E−09
Tyrosine metabolism	map00350	9/42	0.39	1.90E−06
Citrate cycle (TCA cycle)	map00020	8/20	0.38	1.80E−09
Glycerolipid metabolism	map00561	3/16	0.37	5.21E−06
Tryptophan metabolism	map00380	5/41	0.36	5.14E−11
Phenylalanine metabolism	map00360	2/12	0.36	1.90E−06
Glyoxylate and dicarboxylate metabolism	map00630	11/32	0.35	5.65E−07
Starch and sucrose metabolism	map00500	3/15	0.34	1.17E−03
Ascorbate and aldarate metabolism	map00053	2/10	0.25	1.00E−11
Amino sugar and nucleotide sugar metabolism	map00520	6/37	0.21	1.53E−06
Lysine degradation	map00310	8/25	0.19	1.66E−09
Purine metabolism	map00230	17/66	0.18	2.10E−11
Aminoacyl-tRNA biosynthesis	map00970	19/48	0.17	5.48E−13
Glutathione metabolism	map00480	9/28	0.17	1.12E−05
Glycerophospholipid metabolism	map00564	5/36	0.16	1.85E−07
Glycolysis/Gluconeogenesis	map00010	3/26	0.14	3.77E−02
Pentose and glucuronate interconversions	map00040	3/18	0.13	1.00E−11
Pentose phosphate pathway	map00030	2/22	0.13	1.13E−03

Reference pathway library was obtained from KEGG database (Mus musculus, version Oct. 2019).

Pathways including ≥ 2 hits and having impact > 0.1 are listed.

The most impacted pathways included the metabolism of phenylalanine, tyrosine, and tryptophan, which are sources for the production of the major neurotransmitters catecholamine and serotonin. In fact, the neurotransmitters catecholamine, dopamine, noradrenaline, and adrenaline were either not detected or not significantly changed in terms of abundance, even though the upstream levels of phenylalanine, tyrosine, and L-dopa were significantly increased in the AIMD condition (Fig. 1d). Moreover, serotonin was depleted completely, although its source, tryptophan, was increased in this group (Fig. 1e). Importantly, vitamin B6 compounds (pyridoxal, pyridoxamine, pyridoxin, and pyridoxamine phosphate) were completely depleted or significantly decreased in the AIMD group (Fig. 1f). Vitamin B6 is a co-factor required for aromatic L-amino acid decarboxylase (AADC) activity, which catalyzes dopamine and serotonin synthesis. In addition, the levels of taurine were significantly decreased in the AIMD group (Fig. 1g).

Major inhibitory neurotransmitters, namely, glycine and gamma aminobutyric acid (GABA; in alanine, aspartate, and glutamate metabolism), were significantly increased in the AIMD group, whereas acetylcholine was detected in less than half of the mice in each group, and its concentration did not differ significantly between them (Fig. 1h).

### Depletion of gut microbiota changed the sleep/awake architecture

**Figure 2:**
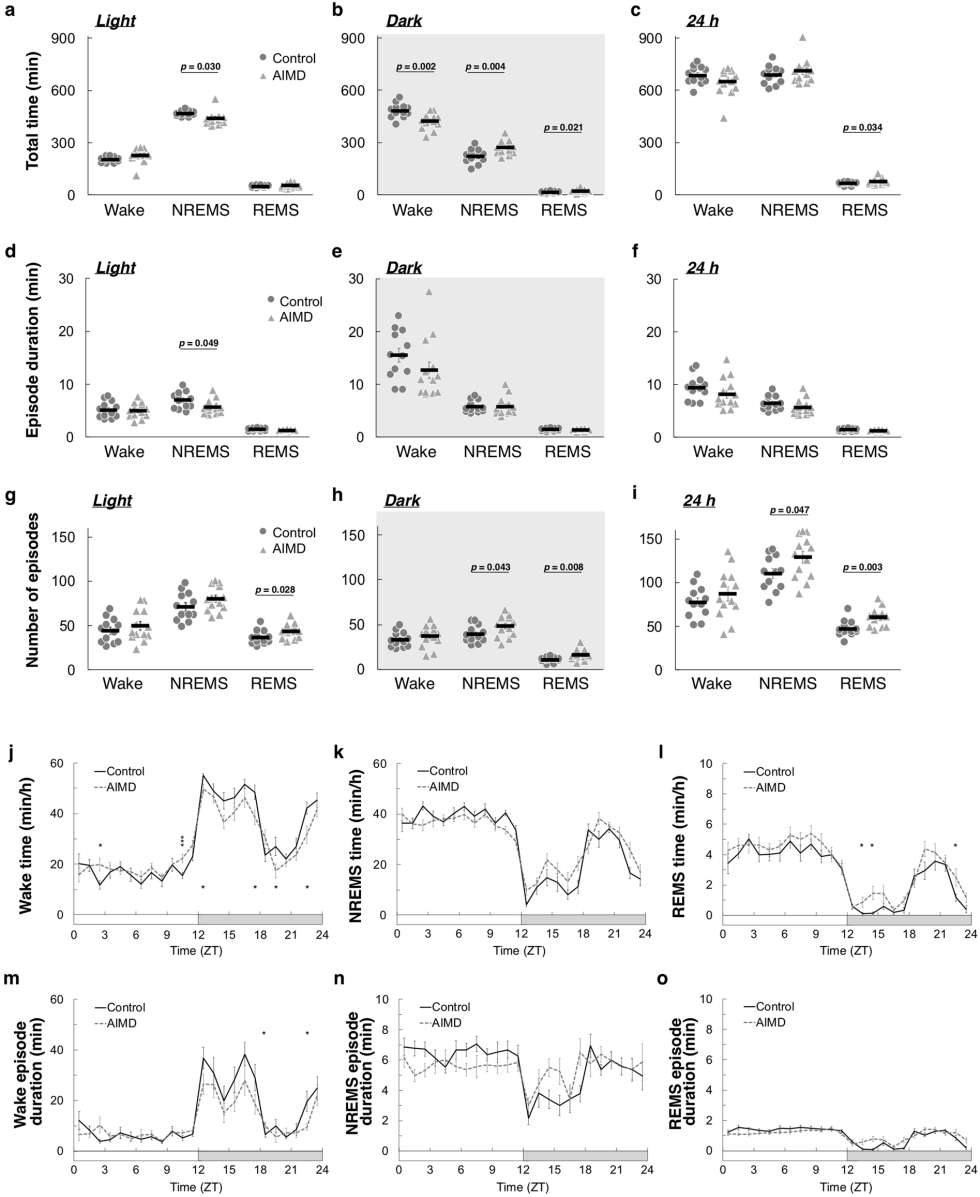
Comparison of sleep/awake architectures between antibiotic-induced microbiota-depleted (AIMD) and control mice. (**a–c**) Total time in the wakefulness, non-rapid eye movement sleep (NREMS), and REMS states in the light phase (**a**), dark phase (**b**), and over 24 h (**c**). (**d–f**) Duration of the wakefulness, NREMS, and REMS episodes in the light phase (**d**), dark phase (**e**), and over 24 h (**f**). (**g–i**) The number of episodes of the wakefulness, NREMS, and REMS states in the light phase (**g**), dark phase (**h**), and over 24 h (**i**). The *circles* and *triangles* represent individual 2-day mean values for the control (*n* = 12) and AIMD (*n* = 13) groups, respectively (**a**–**i**). The *black bars* represent the mean values. (**j**–**l**) Hourly plots of the total times in the wakefulness (**j**), NREMS (**k**), and REMS (**l**) states. (**m**–**o**) Hourly plots of the durations of the wakefulness (**m**), NREMS (**n**), and REMS (**o**) episodes. The *black line* and *gray line* represent the control (*n* = 12) and AIMD (*n* = 13) groups, respectively (**j**–**o**). The *horizontal open and filled bars* on the x-axes indicate the 12 h light and 12 h dark phase, respectively. The possible mean difference was assessed using Welch’s *t* test (**a**–**i**) or two-way ANOVA followed by post hoc two-tailed Welch’s *t* test (**j**–**o**). *** *p* < 0.005; ** *p* < 0.01; * *p* < 0.05. No significant difference in variance was observed (*p* = 0.137 (**k**), *p* = 0.762 (**n**) and *p* = 0.541 (**o**), two-way ANOVA).

Metabolome profiling revealed significant changes in neurotransmission-related intestinal metabolites in mice that had undergone AIMD. The EEG/EMG data for each mouse were analyzed to determine the wakefulness, NREMS, and REMS states. The findings revealed that the AIMD mice spent a significantly shorter time in NREMS during the light phase, from Zeitgeber time (ZT) 0 to 12 (Fig. 2a), whereas they spent a significantly longer time in NREMS and REMS during the dark phase, from ZT 12 to 0, compared with the controls (Fig. 2b). The total time spent in wakefulness was significantly shorter in the AIMD mice than in the controls only during the dark phase (Fig. 2a,b). In 24 h, only the total time spent in REMS was significantly longer in AIMD, whereas the total times spent in the states of wakefulness and NREMS were similar between the two groups (Fig. 2c). The mean durations of the wakefulness, NREMS, and REMS episodes were similar between the AIMD and control groups, except for that of NREMS in the light phase, which was significantly shorter in the AIMD group (Fig. 2d–f). However, the number of wakefulness, NREMS, and REMS episodes tended to increase in the AIMD group in all phases. In particular, the number of REMS episodes was significantly higher throughout the day, and that of NREMS episodes was significantly higher during the dark phase and over 24 h (Fig. 2g–i).

The hourly plots of sleep/wake amounts revealed that the diurnal variation of wakefulness and REMS was significantly different between the AIMD and control groups (*p* = 0.037 for wakefulness and *p* < 0.001 for REMS, two-way analysis of variance (ANOVA)), although the diurnal variation of NREMS was not different significantly (Fig. 2j–l). The time spent in wakefulness was relatively long during the light phase and short throughout the dark phase in the AIMD group (Fig. 2j), whereas that in REMS was relatively long throughout the day in the AIMD compared with the control group (Fig. 2l). Diurnal variations in the episode durations of wakefulness, NREMS and REMS exhibited similar trends with the hourly changes of the total time spent in each state, and only the episode duration of wakefulness was significantly different between the two groups (*p* = 0.005, two-way ANOVA; Fig. 2m–o). That is to say, the changes in time spent in wakefulness were related to the wakefulness episode duration, whereas those in REMS were simply not related to the REMS episode duration.

### REMS occurred more frequently under microbiota depletion

**Figure 3:**
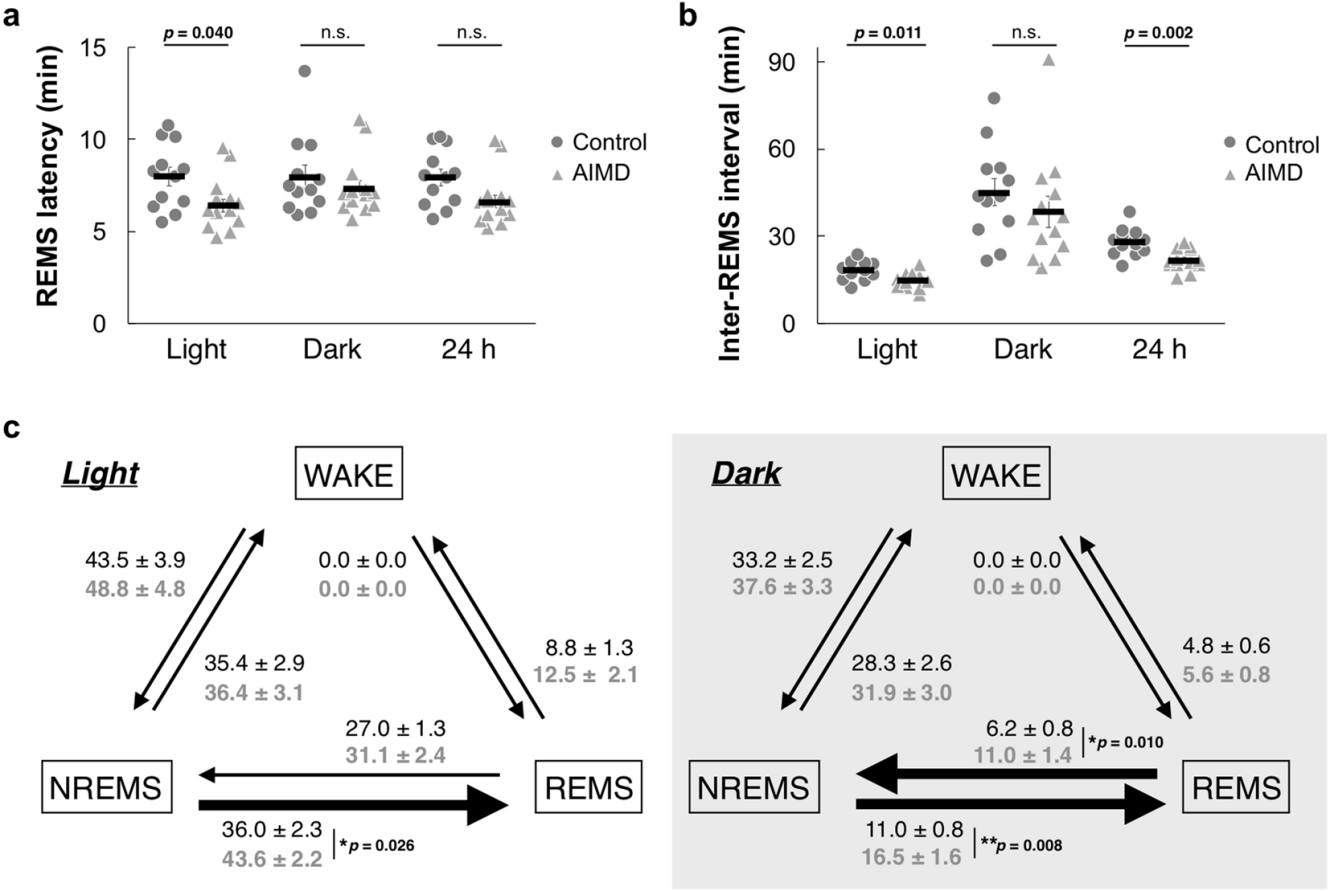
Comparison of phase-transition-related parameters. (**a**,**b**) Rapid eye movement sleep (REMS) latency (**a**) and inter-REMS intervals (**b**) in the light phase, dark phase, and over 24 h. The *circles* and *triangles* represent individual 2-day mean values of the control and antibiotic-induced microbiota-depleted (AIMD) groups, respectively. The *black bars* represent mean values. (**c**) The number of transitions between the wakefulness, non-REMS, and REMS episodes in the light and dark phases. The *black letters in the upper row* and *gray letters in the lower row* represent 2-day mean values ± SEM of the control (*n* = 12) and AIMD (*n* = 13) groups, respectively. Transitions showing significant differences are represented by *thick arrows*. The possible mean difference was assessed using two-tailed Welch’s *t* test. *n.s.* not significant.

The AIMD group displayed abnormal REMS characteristics, so we analyzed the details. REMS latency, which is the duration from the beginning of an NREMS episode to the onset of REMS, tended to be shorter in the AIMD group, although the difference was significant only in the light phase (Fig. 3a). The inter-REMS interval, which is the period between two REMS episodes, also tended to be shorter in the AIMD group throughout the day, although the difference between the groups was significant only during the light phase and over the full 24 h (Fig. 3b). Consequently, REMS occurred more frequently in the AIMD mice than the controls, especially during the light phase.

To consider which direction of transition between the wakefulness, NREMS, and REMS states caused more frequent REMS episodes, the number of transitions between each state was counted (Fig. 3c). Transitions from wakefulness to NREMS, NREMS to wakefulness, and REMS to wakefulness per day occurred in similar frequencies for the AIMD mice and controls. No transition from wakefulness to REMS was detected in either group, indicating that there was no sleep-onset REMS, which is an abnormal behavior. Notably, transition from NREMS to REMS was significantly more frequent during both the light and dark phases in AIMD mice compared with the controls. The transition from REMS to NREMS in the AIMD group occurred significantly more frequently than that in the controls only in the dark phase (Fig. 3c).

### Reduction in REMS theta-spectral power under microbiota depletion

**Figure 4:**
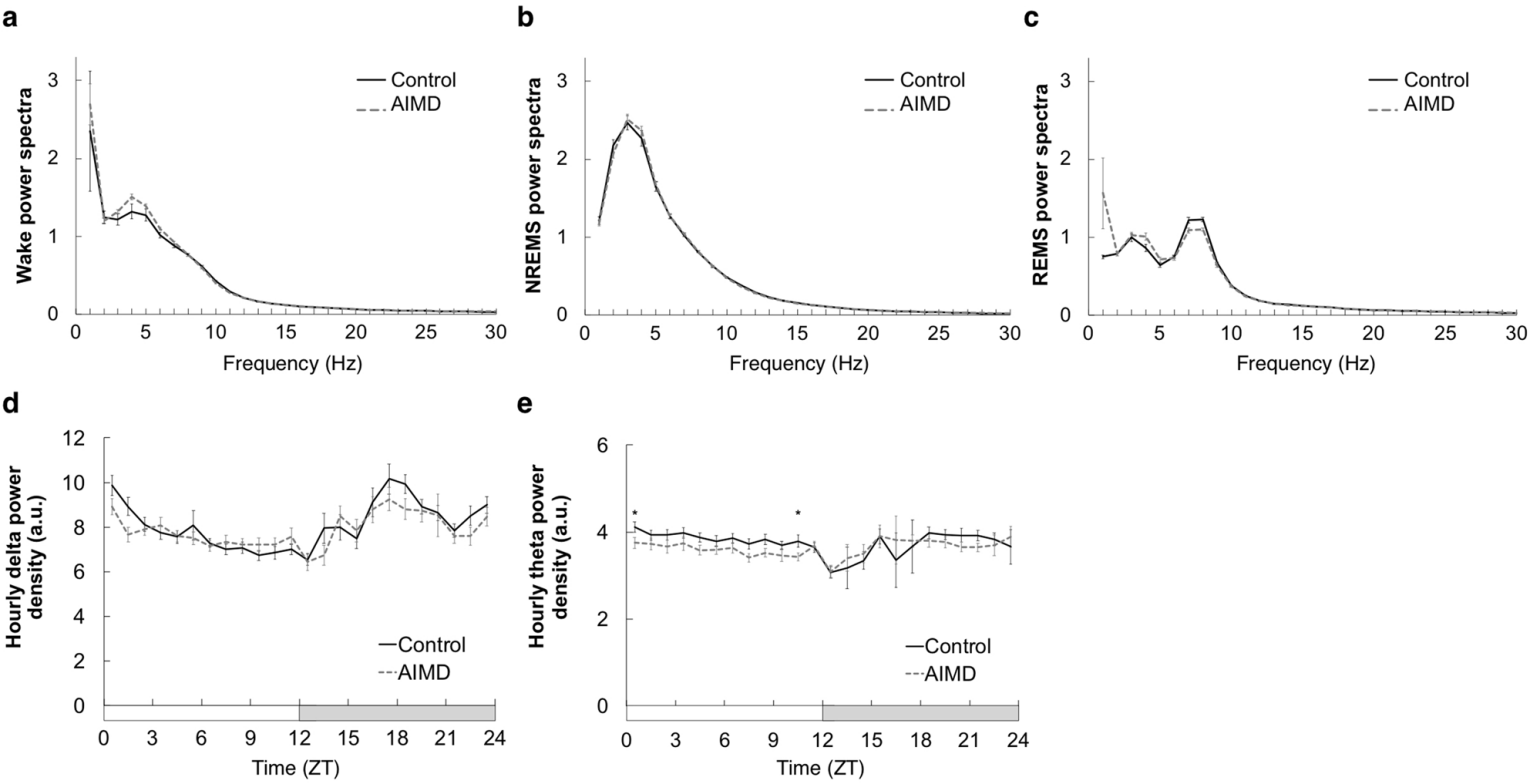
Analysis of the EEG spectra recorded during the wakefulness, non-rapid eye movement sleep (NREMS), and REMS states. (**a**–**c**) Mean relative power spectra of EEG during the wakefulness (**a**), NREMS (**b**), and REMS (**c**) states over 2 days. (**d**,**e**) Hourly changes in delta power density during NREMS (**d**) and theta power density during REMS (**e**). The *black line* and *gray line* represent the control (*n* = 12) and AIMD (*n* = 13) groups, respectively. The *horizontal open and filled bars* on the x-axes indicate the 12 h light and 12 h dark phases, respectively. The possible mean difference was assessed using two-way ANOVA followed by post hoc two-tailed Welch’s *t* test. * *p* < 0.05 (**e**; *p* < 0.001, two-way ANOVA). No significant difference in variance was observed (*p* = 0.330 (**a**), *p* = 0.941 (**b**), *p* = 0.147 (**c**) and *p* = 0.055 (**d**), two-way ANOVA).

Finally, the EEG power spectra recorded during the wakefulness, NREMS, and REMS states were compared. During all the wakefulness, NREMS and REMS phases, there were no significant differences in the EEG power spectra between the AIMD and control groups (Fig. 4a–c). Hourly changes in the delta and theta power densities of the EEG were then calculated (Fig. 4d–e). The delta power density, which is considered to be an indicator of the homeostatic sleep need^17^, during NREMS was not significantly different between the two groups (Fig. 4d). In contrast, the theta power density during REMS was different between the two groups (*p* < 0.001, two-way ANOVA) and significantly low at ZT0 and ZT10 in the AIMD group (Fig. 4e).

## Discussion

The effect of the gut microbiota on sleep/wake regulation has been investigated. Metabolome analysis using CE-TOFMS indicated that, under the AIMD condition, significant changes occurred in neurotransmission-related amino acids and vitamin metabolism in the intestine. Sleep analyses based on EEG/EMG recordings revealed that the AIMD condition affected the NREMS and REMS patterns and REMS spectral properties. The total time spent in NREMS for the AIMD mice was shorter during the light phase (a sleep phase for mice), and longer during the dark phase (an active phase) than that for the controls, indicating that the amplitude of behavioral circadian rhythmicity was reduced. The shorter duration of NREMS episodes during the light phase and more frequent transitions between NREMS and REMS in the AIMD mice suggests that AIMD promotes the fragmentation of NREMS episodes in the light phase. However, the total time spent in wakefulness and NREMS over 24 h was unchanged, indicating that the homeostatic regulation might enable adjustments that result in the requirements for total time in NREMS to be met. Moreover, the sleep need indicated by the delta power density during NREMS was not affected by AIMD.

Serotonin plays a role in the microbiota–host interaction and regulates physiological functions^6,18–21^. Serotonergic signaling is also involved in sleep/wake regulation in the central nervous system (CNS)^22–24^. Under the AIMD condition, serotonin concentrations were found to be significantly reduced in the gut. Serotonin deficiency in the brain causes increases in the total NREMS time during dark phases and decreases in them during light phases while maintaining the necessary total NREMS time over 24 h^25^. This phenotype was consistent with our result, indicating the possibility that serotonin is involved in the communication from the gut microbiota to sleep regulation.

In terms of REMS, an increase in NREMS/REMS transitions resulted in a greater number of REMS episodes and more total time spent in REMS during both light and dark phases in the AIMD mice. This is also consistent with the suggestion that serotonin depletion—induced by dietary tryptophan depletion—causes short latency and prolonged total time in REMS^26^, although the CNS-restricted depletion of serotonergic signaling results in shorter periods spent in REMS^25,27^. These findings suggest that the summary effect of peripheral and central serotonin depletion, which may occur under low-vitamin-B6 conditions, leads to an increase in REMS for the AIMD mice. In fact, changes in the gut microbiome have been associated with the REMS behavior disorder in patients with Parkinson’s disease^28^. Interestingly, the REMS theta power density was reduced in some timepoints during the light phase under the AIMD condition. The reduction in theta power density during all wakefulness, NREMS and REMS was observed with the up-regulation of the glutamate transporter GLT-1 induced by ceftriaxone treatment^29,30^. However, such effects on EEG power spectra during the wakefulness and NREMS including the theta power reduction were not observed in this study, suggesting that the direct effect of antibiotics to brain EEG was relatively small in our experimental condition. Consequently, the microbiota depletion may be related to the reduction of REMS-specific theta power density. The delta power density during NREMS was similar between the AIMD and control mice, indicating that sleep need in the AIMD mice was similar with that in controls under the ad libitum sleep condition. The effect of sleep deprivation on EEG spectra of the AIMD mice should be examined next to investigate the impact of gut microbiome on sleep homeostasis.

Vitamin B6 depletion in AIMD may affect the metabolism of catecholamines, including L-dopa, dopamine, noradrenaline, and adrenaline, which are major neurotransmitters associated with the regulation of arousal. About half of the total peripheral dopamine is produced in the intestinal tract, and the levels of this metabolite are reduced under the AIMD condition^31,32^. Moreover, increased amounts of the inhibitory neurotransmitters GABA and glycine were observed under the AIMD condition. We speculate that the fluctuation in the balance of neurotransmitters may modulate various peripheral physiologies such as gut blood flow, which may possibly affect sleep/wake cycle indirectly^33^. Further studies including metabolome analyses of plasma and specific brain regions are warranted to address the connection between intestinal neurotransmitters and sleep/wake cycle.

Furthermore, the gut microbiota and its metabolites show circadian rhythms^1–3^. Disrupting these oscillations perturbs the host circadian clocks both in peripheral tissues and the hypothalamic area containing the suprachiasmatic nucleus, where the central master clock exists^3,34,35^. Consequently, the gut microbiota could affect the sleep/awake patterns via the circadian clock. The mildly dampened diurnal rhythmicity of sleep/wake cycle in the AIMD mice may occur through the modulation of the circadian clock, although the onset timing of sleep and active phases was normal. Animal models exhibiting circadian arrhythmicity may help separating the function of the circadian clock from sleep regulation by microbiota.

Chronic treatment with a broad-spectrum antibiotic mixture containing ampicillin, vancomycin, neomycin, and metronidazole depletes the gut microbiota and decreases its alpha diversity^6,8^. Compositional alterations to the gut microbiota were detected after > 1 week from the onset of treatment, and 3–4 weeks was enough to stabilize the gut microbiota under the treatment condition^6,36^. Therefore, we considered that 28 days of antibiotic treatment was enough to deplete the gut microbiota. The limitations to the present study include the fact that we could not exclude the direct effect of the antibiotics on brain functions, although known effects of beta-lactam antibiotics such as epileptic seizure were not observed in our study. Germ-free animals could be used to examine the effect of the gut microbiota on sleep regulation without using antibiotics. However, this would pose another challenge because brain development and energy metabolism, which are possible factors that alter sleep/wake characteristics, in germ-free mice are different to those of mice bred in a conventional environment^12,37,38^. Alternatively, the composition of the gut microbiota could be controlled by feeding and inoculation. This would not cause microbiota depletion but would enable insights to be obtained about the function of each intestinal microorganism in regulating sleep.

This study has revealed the relationship between the gut microbiota and sleep/wake regulation, indicating that changes to the intestinal microbiota have the potential to improve sleep-related problems such as daytime sleepiness and insomnia. Notably, some prebiotics have already been shown to improve subjective sleep quality in humans and stress-induced sleep disruption in rats^39,40^. Further study will be required to unveil which metabolites produced by the gut microbiota contribute to sleep regulation.

## Materials and methods

### Animals

All animal procedures were approved by the institutional animal care and use committee of the University of Tsukuba, in compliance with the national guidelines of Japan (approval number 17-323 and 18-164). Mice were maintained according to the institutional guidelines of the animal facilities at the Laboratory of Animal Resource Center, University of Tsukuba, and were kept under a 12-h light/dark cycle, 23 ± 2 °C, and 55 ± 5% humidity with ad libitum food and weakly acidic water with or without antibiotics.

### Antibiotic administration

C57BL/6 male mice were randomized into two groups. The AIMD group (*n* = 13) was housed with access to weakly acidic drinking water containing four types of antibiotic (1 g/L ampicillin sodium, 0.5 g/L vancomycin hydrochloride, 1 g/L neomycin sulfate, and 1 g/L metronidazole; all from Nacalai Tesque Inc., Kyoto, Japan) from 8 weeks of age to deplete their gut microbiota^6,8,14^. As several mice initially exhibited low levels of water consumption, metronidazole was excluded from the drinking water following the surgery to implant electrodes until the end of the experiment. The other group of mice was housed as controls (*n* = 12) with access to weakly acidic drinking water containing no antibiotics.

### Electrode implantation and EEG/EMG recording

Mice were subjected to electrode implantation surgery 10–12 days after they were first given access to the antibiotic–water supply. An electrode for recording EEG/EMG signals was implanted to the scalp of each mouse under isoflurane anesthesia, as described by Miyoshi et al*.*^41^. All mice were allowed 7 days of recovery after surgery in their home cage.

Mice were acclimated to EEG/EMG recording for 8 days individually at 20 days after they were first given access to the antibiotic–water supply. EEG and EMG signals were then recorded for 2 days at 29–31 days after they were first given access to the antibiotic–water supply. The EEG/EMG signals were amplified, filtered (EEG: 0.5–100 Hz; EMG: 0.5–300 Hz) with a multi-channel amplifier (#AB-611J; NIHON KODEN, Tokyo, Japan), and digitized at a sampling rate of 250 Hz using an analog-to-digital converter (#PCI-6220; National Instruments, TX, USA) with LabVIEW software (National Instruments).

### Sleep analysis

The EEG/EMG data were visualized and semi-automatically analyzed using MATLAB-based software, followed by visual inspection. Each 20-s epoch was staged into wakefulness, NREMS, and REMS. The EEG signals were subjected to fast Fourier transform analysis from 1 to 30 Hz with 1-Hz bins using MATLAB-based custom software. The relative power spectra was calculated as a ratio of the EEG power in each frequency bin to the total EEG power over 15–30 Hz frequency bins, which are outside of sleep/wake-related spectra and show relatively small variation through a day, for each wakefulness, NREMS, and REMS state in a 24-h period. The hourly delta power density (a.u.) was defined as the ratio of the mean delta band power (1–4 Hz) in each hour to the mean total EEG power over 15–30 Hz frequency bins in a 24-h period of the NREMS epochs^17^. The hourly theta power density (a.u.) was defined as the ratio of the mean theta band power (6–9 Hz) in each hour to the mean total EEG power over 15–30 Hz frequency bins in a 24-h period of the REMS epochs.

### Feces collection and viable bacteria counting

Feces from the AIMD (*n* = 13) and control (*n* = 12) mice were collected at ZT 0–2 and immediately stored under anaerobic conditions (AnaeroPack-Kenki; Mitsubishi Gas Chemical Co., Ltd., Tokyo, Japan). Here, 20 mg of fecal grains was diluted in 1 mL of PBS, and this solution was diluted 10^3^-, 10^5^-, and 10^7^-times with PBS before culturing on Gifu anaerobic medium (GAM) agar for 24 h under anaerobic conditions at 37 °C. The number of colonies was counted following 24 h of culture to estimate the number of viable bacteria per gram.

### Cecal content collection and preparation for metabolome analysis

The cecum from each of the AIMD (*n* = 13) and control (*n* = 12) mice was collected in a random order at ZT 2–6 on the same day as the feces were collected. The cecal contents were frozen in liquid nitrogen immediately after collection and stored in a freezer at − 80 °C. Metabolite extraction was carried out as described previously, with slight modifications^42^. The metabolites were extracted from 10 mg of freeze-dried cecal contents, which were diluted in 500 µL of methanol containing internal standard compounds [10 µM each of L-methionine sulfone (#502-76641; FUJIFILM Wako Pure Chemical Corporation, Osaka, Japan), D-camphor-10-sulfonic acid (#037-01032; FUJIFILM Wako Pure Chemical Corporation), and 2-(N-morpholino)ethanesulfonic acid (#349-01623; Dojindo Molecular Technologies, Tokyo, Japan)] and subjected to homogenization with 0.1 g of 0.1 mm zirconia beads and four 3.0 mm zirconia beads (BioSpec Products, OK, USA) for 5 min at 1500 rpm using a multi-sample cell homogenizer (Shake Master NEO; Bio-Medical Science, Tokyo, Japan). Chloroform (500 µL) and Milli-Q water (200 µL) were added into each sample tube, homogenized for 5 min at 1500 rpm, and centrifuged for 30 min at 20,000 × *g* at 20 °C. A 300 µL sample of the supernatant was added to a 5-kDa-cutoff filtration column (Ultrafree MC-PLHCC 250/pk for metabolome analysis; Human Metabolome Technologies, Yamagata, Japan) and centrifuged at 9100 × *g* and 4 °C overnight. The filtered solution containing metabolites was concentrated in a centrifugal evaporator for 3 h at 40 °C. The residue was suspended in 50 µL of Milli-Q water containing reference compounds [200 µM each of 3-aminopyrrolidine (#404624; Sigma-Aldrich Inc., MO, USA) and trimesate (#206-03641; FUJIFILM Wako Pure Chemical Corporation)]. Samples were diluted five-fold with Milli-Q water for anion mode measurements and left undiluted for cation mode measurements.

### Metabolome data collection

The metabolites in the sample solutions were detected using CE-TOFMS (Agilent Technologies Inc., CA, USA) in both positive and negative modes, as previously described^15,16,43^. The standard samples containing 20, 50, or 200 µM each of the standard compounds for the positive and negative modes and a blank sample were analyzed, in addition to the experimental samples. Metabolites were identified based on the mass-to-charge ratio (m/z) and migration time of detected peaks using the proprietary software MasterHands version 2.17.3.18 (Keio University, Tsuruoka, Japan). The relative peak area for each metabolite was defined as the absolute peak area for each metabolite normalized by that of the associated internal standard (L-methionine sulfone for the cation mode and D-camphor-10-sulfonic acid for the anion mode). The concentration (µM) of each metabolite in the sample solution was calculated according to the ratio of the relative peak area from the experimental sample to that of the standard sample while considering the degradation ratio of the internal standard in the experimental sample. The concentration (nmol/g) of each metabolite in the cecal contents was calculated using the balance of the concentration following the deduction of the concentration in the blank sample.

### Metabolome data analysis

All detected metabolites were subjected to data analysis. For PCA, data were scaled as mean-centered and divided by the standard deviation of each variable to evaluate the whole picture. Factor loading of the first principal component (PC1) was calculated as the Pearson product-moment correlation coefficient (*r*) between the metabolite concentration and the PC1 factor. Functional annotation of metabolites was performed using pathway enrichment analysis according to the MSEA method by utilizing statistical values calculated by quantitative enrichment analysis, based on the Global Test, using concentration values without any scaling. The metabolic pathway library of *Mus musculus* in the KEGG database (version Oct. 2019) was used as the reference^44^. Pathway topology was analyzed with relative betweenness centrality by focusing on the global network topology. The pathway impact value was calculated as the cumulative percentage of the matched metabolite nodes. PCA and pathway enrichment analysis were conducted using the web-based application MetaboAnalyst for MSEA^45^.

### Statistics

Data were presented as mean ± standard error of mean (SEM). Statistical analyses were performed using R version 3.5.1 (The R Foundation for Statistical Computing)^46^ and Microsoft Excel 365 (Microsoft Corporation). The possible mean difference between the AIMD and control groups was assessed using one-way ANOVA followed by post hoc Benjamini–Hochberg false discovery rate correction for metabolome profiling, two-way ANOVA followed by post hoc two-tailed Welch’s *t* test for time series data and EEG power spectra, or two-tailed Welch’s *t* test. In all cases, *p* values lower than 0.05 were considered significant.

## Supplementary information

Supplementary Tables.

Supplementary Table S4.

## Data Availability

The data for this study is available in the section of supplementary information (Supplementary Table S4 online).
