# Supplementary material for: Gut microbiota depletion by chronic antibiotic treatment alters the sleep/wake architecture and sleep EEG power spectra in mice
Source: Sci Rep. 2020 Nov 11;10:19554. doi: 10.1038/s41598-020-76562-9 (PMC7659342; doi:10.1038/s41598-020-76562-9)
Supplement: Supplementary file 1 — Supplementary Tables. [file 41598_2020_76562_MOESM1_ESM.pdf]

Supplementary information

Gut microbiota depletion by chronic antibiotic treatment alters the sleep/wake architecture and sleep EEG power spectra in mice

Yukino Ogawa[1,2,3], Chika Miyoshi[1], Nozomu Obana[4], Kaho Yajima[2], Noriko Hotta-Hirashima[1], Aya Ikkyu[1], Satomi Kanno[1], Tomoyoshi Soga[2], Shinji Fukuda[2,4-6*], Masashi Yanagisawa[1,7-9*]

1. International Institute for Integrative Sleep Medicine (WPI-IIIS), University of Tsukuba, 1-1-1 Tennodai, Tsukuba, Ibaraki 305-8575, JAPAN
2. Institute for Advanced Biosciences, Keio University, 246-2 Mizukami, Kakuganji, Tsuruoka, Yamagata 997-0052, JAPAN
3. Food Research Institute, National Agriculture and Food Research Organization (NARO), 2-1-12 Kannondai, Tsukuba, Ibaraki 305-8642, JAPAN
4. Transborder Medical Research Center, University of Tsukuba, 1-1-1 Tennodai, Tsukuba, Ibaraki 305-8575, JAPAN
5. Intestinal Microbiota Project, Kanagawa Institute of Industrial Science and Technology, 3-25-13 Tonomachi, Kawasaki-ku, Kawasaki, Kanagawa 210-0821, JAPAN
6. Metabologenomics, Inc., 246-2 Mizukami, Kakuganji, Tsuruoka, Yamagata 997-0052, JAPAN
7. Life Science Center for Survival Dynamics, Tsukuba Advanced Research Alliance (TARA), University of Tsukuba, 1-1-1 Tennodai, Tsukuba, Ibaraki 305-8575, JAPAN
8. R&D Center for Frontiers of Mirai in Policy and Technology (F-MIRAI), University of Tsukuba, 1-1-1 Tennodai, Tsukuba, Ibaraki 305-8575, JAPAN
9. Department of Molecular Genetics, University of Texas Southwestern Medical Center, Dallas, Texas 75390-8584, USA

* Corresponding authors.

Table S1. Metabolites in cecal contents detected only in the control mice.

Metabolites	KEGG ID	Concentration (nmol/g)		Number of detected samples		*p* value	*r* value
		Control	AIMD	Control	AIMD		(correlation with PC1)
6-Hydroxyhexanoate	C06103	153.4 ± 6.2	0	12	0	<0.001	0.981
Alpha-Methylserine	C02115	7.5 ± 0.4	0	12	0	<0.001	0.979
Desaminotyrosine	C01744	1619.6 ± 108.1	0	12	0	<0.001	0.972
Propionate	C00163	42602.1 ± 2328.0	0	12	0	<0.001	0.966
4-Hydroxymethylimidazole	C05562	4.0 ± 0.3	0	12	0	<0.001	0.963
Pentanoate	C00803	2931.6 ± 199.7	0	12	0	<0.001	0.953
3-Phenylpropionate	C05629	1006.2 ± 93.3	0	12	0	<0.001	0.949
Glutarate	C00489	344.7 ± 34.2	0	12	0	<0.001	0.946
Pyridoxal	C00250	12.4 ± 0.6	0	12	0	<0.001	0.943
Serotonin	C00780	13.7 ± 1.2	0	12	0	<0.001	0.942
N-Acetyl-glucosamine 1-phosphate	C04501	162.7 ± 16.0	0	12	0	<0.001	0.941
CMP	C00055	47.4 ± 4.2	0	12	0	<0.001	0.939
6-Aminohexanoate	C02378	26.1 ± 2.4	0	12	0	<0.001	0.937
dTMP	C00364	69.8 ± 7.5	0	12	0	<0.001	0.935
Pimelate	C02656	46.1 ± 5.0	0	12	0	<0.001	0.933
p-Aminobenzoate	C00568	18.8 ± 2.0	0	12	0	<0.001	0.917
Pyridoxine	C00314	11.4 ± 1.3	0	12	0	<0.001	0.910
5-Aminolevulinate	C00430	8.2 ± 1.1	0	12	0	<0.001	0.904
Thiamine monophosphate	C01081	14.3 ± 1.7	0	12	0	<0.001	0.904
N-methyl-L-glutamic Acid	C01046	10.8 ± 1.4	0	12	0	<0.001	0.903
Pyridoxamine 5'-phosphate	C00647	13.8 ± 1.6	0	12	0	<0.001	0.902
Phosphorylcholine	C00588	46.7 ± 5.3	0	12	0	<0.001	0.895
Tyramine	C00483	4.6 ± 0.5	0	12	0	<0.001	0.888
3-Methylbutanoate	C08262	1059.5 ± 95.9	0	12	0	<0.001	0.880
2-Isopropylmalate	C02504	9.1 ± 1.3	0	12	0	<0.001	0.870
N-Methylalanine	C02721	91.0 ± 13.1	0	12	0	<0.001	0.868
D-Ribulose 5-phosphate	C00199	68.7 ± 9.4	0	12	0	<0.001	0.867
UMP	C00105	81.3 ± 11.1	0	12	0	<0.001	0.867
NMN	C00455	155.4 ± 23.0	0	12	0	<0.001	0.856
D-Sedoheptulose 7-phosphate	C05382	140.6 ± 23.4	0	12	0	<0.001	0.851
N-Acetylaspartate	C01042	228.5 ± 25.7	0	12	0	<0.001	0.848
N1,N12-Diacetylspermine	C03413	6.8 ± 1.2	0	12	0	<0.001	0.842
AMP	C00020	172.4 ± 28.6	0	12	0	<0.001	0.842
6-Hydroxynicotinate	C01020	56.4 ± 9.0	0	12	0	<0.001	0.840
Glycerophosphate	C00093	430.3 ± 76.6	0	12	0	<0.001	0.830
2,4-Diaminobutyrate	C03283	5.7 ± 0.9	0	12	0	<0.001	0.817
Isopropanolamine	C05771	28.3 ± 4.9	0	12	0	<0.001	0.816
Cytosine	C00380	4.5 ± 0.6	0	12	0	<0.001	0.805
Deoxycholic acid	C04483	2638.1 ± 348.6	0	12	0	<0.001	0.793
D-Glucose 1-phosphate	C00103	46.8 ± 10.5	0	12	0	<0.001	0.767
NAD+	C00003	229.0 ± 50.9	0	12	0	<0.001	0.755
2-Oxoglutarate	C00026	266.9 ± 54.7	0	12	0	<0.001	0.715
Glucose 6-phosphate	C00092	394.4 ± 108.7	0	12	0	<0.001	0.702
Cholate	C00695	1783.9 ± 323.5	0	12	0	<0.001	0.702
Adenine	C00147	52.3 ± 13.2	0	12	0	<0.001	0.591
2-Deoxyglucose 6-phosphate	C06369	34.8 ± 6.2	0	10	0	<0.001	0.812
2-Aminophenol	C01987	1.3 ± 0.3	0	9	0	<0.001	0.783
o-Hydroxybenzoate	C00805	30.8 ± 6.5	0	9	0	<0.001	0.778
Trimethylamine	C00565	196.7 ± 91.6	0	9	0	0.035	0.518
p-Hydroxyphenylacetate	C00642	254.6 ± 58.5	0	8	0	<0.001	0.670
trans-4-Hydroxy-3-methoxycinnamate	C01494	23.3 ± 6.0	0	7	0	<0.001	0.645
3-Ureidopropionate	C02642	30.5 ± 8.7	0	7	0	0.001	0.602
3-Phosphoglyceric acid	C00197	16.9 ± 5.6	0	6	0	0.004	0.629
2,5-Dihydroxybenzoate	C00628	25.7 ± 8.8	0	6	0	0.006	0.601
ADP	C00008	17.5 ± 6.6	0	6	0	0.011	0.588
FAD	C00016	34.0 ± 14.6	0	4	0	0.023	0.496
4-Amino-3-hydroxybutyrate	C03678	1.0 ± 0.4	0	4	0	0.024	0.460
N-Acetylglucosamine 6-phosphate	C00357	8.0 ± 3.7	0	4	0	0.032	0.458
UDP-N-acetylglucosamine	C00043	21.2 ± 9.7	0	4	0	0.032	0.416
5'-Deoxyadenosine	C05198	1.7 ± 0.7	0	4	0	0.023	0.397
1-Methylhistamine	C05127	0.5 ± 0.2	0	4	0	0.029	0.392

Concentration data represent mean ± SEM of 12 and 13 samples of control and AIMD mice, respectively.
p values were calculated by Student's *t* test.
r values represent Pearson's correlation coefficient between concentration and PC1 factor value of PCA analysis.

Table S2. Metabolites in cecal contents detected only in the AIMD mice.

Metabolites	KEGG ID	Concentration (nmol/g)		Number of detected samples		*p* value	*r* value (correlation with PC1)
		Control	AIMD	Control	AIMD		
Ophthalmic acid	-	0	277.6 ± 7.8	0	13	<0.001	-0.984
7-Methylguanine	C02242	0	20.4 ± 0.6	0	13	<0.001	-0.979
Cystathionine	C02291	0	11.7 ± 0.5	0	13	<0.001	-0.975
N-alpha,N-alpha-Dimethylhistidine	C04259	0	8.4 ± 0.4	0	13	<0.001	-0.975
Thymidine	C00214	0	702.7 ± 33.1	0	13	<0.001	-0.971
Glucosaminate	C03752	0	22.8 ± 1.1	0	13	<0.001	-0.967
Oxidized glutathione	C00127	0	5.7 ± 0.3	0	13	<0.001	-0.960
N-Acetyl-b-glucosaminylamine	C01239	0	23.9 ± 1.3	0	13	<0.001	-0.958
N-Acetylvaline	-	0	14.0 ± 0.7	0	13	<0.001	-0.956
Glycerophosphorylcholine	C00670	0	40.2 ± 2.6	0	13	<0.001	-0.956
1-Methyladenosine	C02494	0	18.8 ± 1.2	0	13	<0.001	-0.955
Guanidinosuccinate	C03139	0	14.8 ± 0.9	0	13	<0.001	-0.955
gamma-Glu-cys	C00669	0	34.1 ± 1.8	0	13	<0.001	-0.955
Mucate	C00879	0	177.6 ± 11.8	0	13	<0.001	-0.954
Trigonelline	C01004	0	13.4 ± 0.9	0	13	<0.001	-0.952
Cysteine sulfinate	C00606	0	92.8 ± 6.5	0	13	<0.001	-0.951
2'-Deoxyinosine	C05512	0	510.5 ± 31.1	0	13	<0.001	-0.950
Glucuronate	C00191	0	3647.6 ± 256.6	0	13	<0.001	-0.941
Saccharate	C00818	0	363.1 ± 30.7	0	13	<0.001	-0.930
Kynurenine	C00328	0	6.7 ± 0.6	0	13	<0.001	-0.924
Trimethylamine N-oxide	C01104	0	37.6 ± 3.2	0	13	<0.001	-0.923
2'-Deoxycytidine	C00881	0	81.4 ± 7.3	0	13	<0.001	-0.912
1-Methylnicotinamide	C02918	0	11.8 ± 1.1	0	13	<0.001	-0.912
5-Methyl-2'-deoxycytidine	C03592	0	10.0 ± 0.9	0	12	<0.001	-0.910
Isocitrate	C00311	0	23.3 ± 2.5	0	13	<0.001	-0.884
Urea	C00086	0	14443.9 ± 1392.3	0	13	<0.001	-0.878
2-Quinolinecarboxylate	C06325	0	48.5 ± 6.7	0	11	<0.001	-0.825
Cysteineglutathione disulfide	-	0	9.6 ± 1.5	0	13	<0.001	-0.823
Mannosamine	C03570	0	68.7 ± 9.2	0	13	<0.001	-0.817
7,8-Dihydrobiopterin	C02953	0	7.5 ± 1.7	0	8	<0.001	-0.640
Betonicine	C08269	0	2.2 ± 0.7	0	7	0.003	-0.581
DOPA	C00355	0	4.6 ± 1.7	0	5	0.017	-0.497

Concentration data represent mean ± SEM of 12 and 13 samples of control and AIMD mice, respectively.
p values were calculated by Student's *t* test.
r values represent Pearson's correlation coefficient between concentration and PC1 factor value of PCA analysis.

Table S3. Metabolites in cecal contents detected in both the AIMD and control mice.

Metabolites	KEGG ID	Concentration (nmol/g)		Number of detected samples		Fold change	*p* value	*r* value
		Control	AIMD	Control	AIMD	(AIMD/Control)		(correlation with PC1)
Increased								
L-Proline	C00148	500.7 ± 37.7	19833.3 ± 588.2	12	13	39.6	<0.001	-0.985
Saccharopine	C00449	10.9 ± 1.1	519.2 ± 12.1	12	13	47.5	<0.001	-0.980
Creatinine	C00791	24.9 ± 5.9	1012.1 ± 44.9	12	13	40.6	<0.001	-0.979
Hydroxyproline	C01015	218.3 ± 21.9	1541.7 ± 51.5	12	13	7.1	<0.001	-0.974
Thymine	C00178	177.0 ± 16.4	3817.8 ± 118.5	12	13	21.6	<0.001	-0.972
N-Acetylhistidine	C02997	11.3 ± 1.4	59.2 ± 2.0	12	13	5.2	<0.001	-0.972
gamma-Guanidinobutyrate	C01035	4.9 ± 0.4	101.6 ± 4.5	12	13	20.6	<0.001	-0.970
Urate	C00366	71.1 ± 8.1	394.1 ± 15.2	12	13	5.5	<0.001	-0.969
Thr	C00188	837.5 ± 60.3	14282.6 ± 880.2	12	13	17.1	<0.001	-0.960
Gly-Leu	C02155	31.2 ± 1.7	64.2 ± 1.5	12	13	2.1	<0.001	-0.959
Allantoin	C01551	285.4 ± 20.6	1064.2 ± 45.2	12	13	3.7	<0.001	-0.948
Arg	C00062	510.4 ± 79.7	4795.4 ± 290.7	12	13	9.4	<0.001	-0.948
Anserine	C01262	7.1 ± 0.3	18.4 ± 0.8	12	13	2.6	<0.001	-0.925
Carnitine	C00318	43.6 ± 4.7	137.3 ± 5.8	12	13	3.1	<0.001	-0.923
Malonate	C00383	56.3 ± 8.9	527.2 ± 37.6	12	13	9.4	<0.001	-0.914
Guanosine	C00387	56.9 ± 3.9	580.2 ± 46.5	12	13	10.2	<0.001	-0.913
Cytidine	C00475	24.2 ± 2.2	200.3 ± 16.9	12	13	8.3	<0.001	-0.909
Lys	C00047	1883.7 ± 128.1	6882.7 ± 459.5	12	13	3.7	<0.001	-0.908
Argininosuccinate	C03406	16.4 ± 2.1	112.0 ± 8.0	12	13	6.8	<0.001	-0.905
Gly	C00037	1427.2 ± 95.4	8850.4 ± 862.7	12	13	6.2	<0.001	-0.892
L-Glutamine	C00064	1202.5 ± 187.3	8406.9 ± 809.1	12	13	7.0	<0.001	-0.887
Asn	C00152	161.2 ± 34.8	6669.4 ± 784.0	12	13	41.4	<0.001	-0.883
Leu	C00123	443.2 ± 45.3	2178.2 ± 200.6	12	13	4.9	<0.001	-0.881
N6,N6,N6-Trimethyl-L-lysine	C03793	18.1 ± 1.3	33.8 ± 1.0	12	13	1.9	<0.001	-0.881
Ser	C00065	729.4 ± 31.1	6160.1 ± 663.5	12	13	8.4	<0.001	-0.880
Gly-Gly	C02037	7.0 ± 0.6	38.0 ± 3.3	12	13	5.4	<0.001	-0.876
His	C00135	221.0 ± 20.5	1476.3 ± 157.2	12	13	6.7	<0.001	-0.872
Ile	C00407	271.2 ± 20.6	1586.3 ± 164.1	12	13	5.8	<0.001	-0.870
Creatine	C00300	884.2 ± 155.4	2981.0 ± 146.5	12	13	3.4	<0.001	-0.863
Phe	C00079	199.7 ± 16.8	935.5 ± 94.6	12	13	4.7	<0.001	-0.862
Val	C00183	632.1 ± 63.9	3364.8 ± 353.0	12	13	5.3	<0.001	-0.857
Trp	C00078	75.8 ± 5.1	263.2 ± 25.3	12	13	3.5	<0.001	-0.856
Choline	C00114	276.4 ± 31.2	1835.2 ± 202.7	12	13	6.6	<0.001	-0.836
Tyr	C00082	401.5 ± 32.2	1441.3 ± 156.7	12	13	3.6	<0.001	-0.827
ADMA	C03626	33.0 ± 3.2	64.1 ± 2.3	12	13	1.9	<0.001	-0.823
N-Acetylneuraminate	C00270	165.4 ± 29.9	388.6 ± 23.8	12	13	2.4	<0.001	-0.809
Proline betaine	C10172	8.2 ± 0.6	14.2 ± 0.6	12	13	1.7	<0.001	-0.806
Urocanate	C00785	59.1 ± 11.5	140.4 ± 7.6	12	13	2.4	<0.001	-0.800
o-Acetylcarnitine	C02571	22.0 ± 3.4	51.3 ± 3.4	12	13	2.3	<0.001	-0.786
GABA	C00334	54.1 ± 1.9	133.8 ± 12.8	12	13	2.5	<0.001	-0.752
Betaine	C00719	91.6 ± 10.9	203.2 ± 14.4	12	13	2.2	<0.001	-0.743
Xanthine	C00385	442.5 ± 58.3	827.2 ± 38.1	12	13	1.9	<0.001	-0.719
SDMA	-	6.3 ± 0.4	9.5 ± 0.5	12	13	1.5	<0.001	-0.697
Uridine	C00299	605.4 ± 29.8	957.9 ± 68.8	12	13	1.6	<0.001	-0.681
Glucosamine	C00329	44.5 ± 9.8	203.5 ± 30.4	12	13	4.6	<0.001	-0.674
Ala	C00041	2617.5 ± 214.7	4470.8 ± 451.6	12	13	1.7	0.001	-0.639
Citrate	C00158	64.1 ± 11.0	106.9 ± 6.9	12	13	1.7	0.003	-0.544
Isethionate	C05123	81.4 ± 7.4	115.2 ± 8.1	12	13	1.4	0.005	-0.595
5-Hydroxylysine	C16741	11.7 ± 1.0	30.2 ± 6.0	12	13	2.6	0.008	-0.485
5-Oxoproline	C01879	265.5 ± 14.7	324.8 ± 17.7	12	13	1.2	0.018	-0.559
Glu-Glu	C01425	4.4 ± 0.4	5.8 ± 0.4	12	13	1.3	0.022	-0.409
Met	C00073	249.5 ± 27.3	339.7 ± 26.5	12	13	1.4	0.026	-0.422
N-Acetyl-beta-alanine	C01073	20.7 ± 4.6	330.9 ± 24.4	10	13	16.0	<0.001	-0.934
Xanthosine	C01762	25.4 ± 7.8	125.7 ± 10.5	7	13	5.0	<0.001	-0.827
2'-Deoxyguanosine	C00330	2.8 ± 1.3	206.5 ± 13.2	4	13	72.6	<0.001	-0.948
Cysteine-S-sulfate	C05824	6.8 ± 3.7	572.2 ± 33.4	3	13	83.7	<0.001	-0.966
Threonate	C01620	25.3 ± 17.8	467.4 ± 21.3	2	13	18.5	<0.001	-0.956
Guanidinoacetate	C00581	1.0 ± 0.7	10.0 ± 0.6	2	13	10.3	<0.001	-0.914
Histidinol	C00860	0.6 ± 0.4	5.4 ± 0.2	2	13	8.3	<0.001	-0.856
Cystine	C00491	0.8 ± 0.5	199.8 ± 34.9	2	13	263.7	<0.001	-0.747
Allantoate	C00499	6.3 ± 6.3	175.7 ± 20.7	1	13	27.8	<0.001	-0.833
Quinate	C00296	11.0 ± 4.9	389.9 ± 34.8	4	12	35.4	<0.001	-0.916
2,3-Pyridinedicarboxylate	C03722	11.4 ± 3.2	22.0 ± 3.1	7	11	1.9	0.025	-0.463
Decreased								
5-Hydroxyindoleacetate	C05635	1437.4 ± 113.2	106.7 ± 3.8	12	13	0.074	<0.001	0.961
5-Aminovalerate	C00431	2547.1 ± 165.7	34.9 ± 7.6	12	13	0.014	<0.001	0.953
Azelate	C08261	257.0 ± 13.2	84.0 ± 4.2	12	13	0.327	<0.001	0.944
5-Methylthioadenosine	C00170	31.9 ± 3.2	3.8 ± 0.2	12	13	0.120	<0.001	0.917
SAM+	C00019	39.2 ± 4.2	4.9 ± 0.3	12	13	0.126	<0.001	0.916
gamma-Butyrobetaine	C01181	134.4 ± 8.6	12.2 ± 5.2	12	13	0.090	<0.001	0.907
Homoserine	C00263	95.2 ± 11.1	8.9 ± 0.6	12	13	0.094	<0.001	0.899
3-Methylhistidine	C01152	25.6 ± 2.0	8.3 ± 0.5	12	13	0.324	<0.001	0.891
Hypoxanthine	C00262	1268.7 ± 136.8	235.3 ± 24.9	12	13	0.185	<0.001	0.873
L-Alpha-aminobutyric acid	C02356	124.2 ± 16.1	4.9 ± 0.4	12	13	0.039	<0.001	0.853
Succinate	C00042	1536.7 ± 147.0	125.8 ± 12.4	12	13	0.082	<0.001	0.850
Citrulline	C00327	557.6 ± 39.0	189.9 ± 17.7	12	13	0.341	<0.001	0.845
N-Acetylornithine	C00437	17.0 ± 1.2	8.7 ± 0.3	12	13	0.515	<0.001	0.838
N-epsilon-Acetyllysine	C02727	25.8 ± 2.2	9.2 ± 0.5	12	13	0.355	<0.001	0.832
Nicotinamide	C00153	78.2 ± 10.2	21.2 ± 0.8	12	13	0.271	<0.001	0.831

Table S3. Metabolites in cecal contents detected in both the AIMD and control mice (continued).

Metabolites	KEGG ID	Concentration (nmol/g) Control		Number of detected samples Control	AIMD	Fold change (AIMD/Control)	*p* value	*r* value (correlation with PC1)
		Control	AIMD	Control	AIMD			
Decreased								
Spermidine	C00315	102.9 ± 14.4	14.8 ± 1.4	12	13	0.144	<0.001	0.830
Uracil	C00106	487.8 ± 54.3	175.3 ± 5.9	12	13	0.359	<0.001	0.818
Ectoine	C06231	7.0 ± 0.7	3.3 ± 0.2	12	13	0.478	<0.001	0.797
Glycerol	C00116	10241.8 ± 511.8	6592.4 ± 497.3	12	13	0.644	<0.001	0.794
Taurine	C00245	4361.0 ± 608.9	564.6 ± 27.3	12	13	0.129	<0.001	0.782
Inosine	C00294	1789.7 ± 176.1	808.8 ± 45.5	12	13	0.452	<0.001	0.770
Beta-Alanine	C00099	176.1 ± 16.5	97.8 ± 3.8	12	13	0.555	<0.001	0.763
N1,N8-Diacetylspermidine	-	16.2 ± 3.2	2.1 ± 0.6	12	13	0.132	<0.001	0.744
N1-Acetylspermidine	C00612	69.7 ± 11.8	21.6 ± 2.7	12	13	0.309	<0.001	0.730
Pyridoxamine	C00534	5.6 ± 0.5	2.5 ± 0.2	12	13	0.450	<0.001	0.724
Sarcosine	C00213	77.6 ± 14.8	5.9 ± 0.3	12	13	0.076	<0.001	0.700
Glu	C00025	36348.8 ± 2438.8	23091.1 ± 1578.9	12	13	0.635	<0.001	0.676
Ornithine	C00077	134.3 ± 18.1	60.2 ± 8.0	12	13	0.448	0.001	0.674
Adenosine	C00212	56.3 ± 13.2	16.0 ± 1.5	12	13	0.285	0.005	0.495
Dodecanoate	C02679	62.3 ± 3.9	30.0 ± 3.9	12	11	0.481	<0.001	0.750
Pipecolate	C00408	52.7 ± 6.9	2.8 ± 1.1	12	10	0.054	<0.001	0.875
Pantothenate	C00864	43.8 ± 3.8	13.6 ± 3.0	12	9	0.311	<0.001	0.807
3-Aminoisobutyrate	C05145	11.6 ± 1.5	2.2 ± 0.4	12	9	0.185	<0.001	0.795
Ala-Ala	C00993	130.3 ± 11.9	9.7 ± 4.2	12	8	0.074	<0.001	0.919
N,N-Dimethylglycine	C01026	38.1 ± 4.8	3.5 ± 0.9	12	8	0.093	<0.001	0.825
Hexanoate	C01585	91.0 ± 10.7	10.2 ± 2.8	12	7	0.113	<0.001	0.880
Glycolate	C00160	825.4 ± 98.3	135.0 ± 43.8	12	6	0.164	<0.001	0.834
SAH	C00021	3.5 ± 0.4	0.7 ± 0.3	12	5	0.209	<0.001	0.831
2-Hydroxyglutarate	C02630	785.5 ± 128.6	9.5 ± 3.6	12	5	0.012	<0.001	0.824
Sebacate	C08277	64.9 ± 3.7	1.3 ± 0.6	12	4	0.020	<0.001	0.955
2-Hydroxypentanoate	-	50.8 ± 3.3	3.2 ± 1.4	12	4	0.063	<0.001	0.954
Nicotinate	C00253	955.8 ± 80.1	27.2 ± 12.8	12	4	0.028	<0.001	0.937
4-Pyridoxate	C00847	38.9 ± 1.5	1.5 ± 0.8	12	3	0.038	<0.001	0.980
N-Acetylglutamate	C00624	378.5 ± 27.8	4.1 ± 2.3	12	3	0.011	<0.001	0.924
Decanoate	C01571	29.0 ± 3.8	4.0 ± 2.1	12	3	0.138	<0.001	0.793
Dodecanedioate	C02678	153.3 ± 7.0	0.5 ± 0.4	12	2	0.003	<0.001	0.974
Cysteate	C00506	66.7 ± 7.3	1.4 ± 1.4	12	1	0.021	<0.001	0.913
3-Hydroxy-3-methylglutarate	C03761	10.2 ± 1.5	1.7 ± 0.9	11	3	0.162	<0.001	0.737
4-Acetylbutyrate	C02129	15.4 ± 2.2	6.0 ± 2.0	10	6	0.387	0.004	0.536
Octanoate	C06423	18.1 ± 2.5	5.0 ± 2.2	10	4	0.274	0.001	0.652
Adipate	C06104	20.9 ± 4.1	4.0 ± 2.1	9	3	0.190	0.001	0.660
Glycerate	C00258	355.8 ± 64.5	77.1 ± 42.7	9	3	0.217	0.001	0.542
N-Acetylmuramate	C02713	23.4 ± 7.9	2.4 ± 2.4	7	1	0.102	0.015	0.480
No significant difference								
Asp	C00049	5557.5 ± 492.6	6912.7 ± 457.1	12	13	1.2	0.055	-0.436
Guanine	C00242	46.1 ± 5.1	33.4 ± 3.8	12	13	0.73	0.056	0.379
Methionine sulfoxide	C02989	87.0 ± 9.0	114.0 ± 10.6	12	13	1.3	0.068	-0.456
Phthalate	C01606	8.3 ± 0.5	7.4 ± 0.5	12	13	0.89	0.220	0.249
alpha-Aminoadipate	C00956	35.7 ± 4.8	30.1 ± 1.0	12	13	0.84	0.250	0.348
Thiamine	C00378	16.4 ± 3.1	20.1 ± 1.0	12	13	1.2	0.257	-0.163
N-Acetylglucosamine	C00140	399.9 ± 34.0	436.0 ± 17.1	12	13	1.1	0.341	-0.166
Malate	C00711	198.6 ± 14.0	164.4 ± 37.3	12	13	0.83	0.415	0.192
N8-Acetylspermidine	C01029	6.6 ± 0.8	4.9 ± 2.2	12	13	0.74	0.476	0.204
3-Hydroxybutyrate	C01089	376.3 ± 190.4	285.3 ± 30.0	12	13	0.76	0.628	0.193
Putrescine	C00134	17.0 ± 2.5	14.5 ± 8.0	12	13	0.85	0.775	0.099
Indole-3-ethanol	C00955	33.6 ± 1.8	33.1 ± 1.1	12	13	0.98	0.797	0.086
Hypotaurine	C00519	42.4 ± 5.6	41.0 ± 2.3	12	13	0.97	0.805	0.119
N-Acetylputrescine	C02714	8.9 ± 1.2	8.1 ± 3.5	12	13	0.91	0.829	0.078
Lactate	C00186	4200.8 ± 1607.4	3884.7 ± 269.4	12	13	0.92	0.842	0.027
Diethanolamine	C06772	14.8 ± 2.5	11.8 ± 3.1	10	9	0.80	0.467	0.142
N-Acetylmethionine	C02712	31.1 ± 5.8	39.2 ± 3.7	9	13	1.3	0.247	-0.240
Taurocholate	C05122	515.5 ± 336.6	311.1 ± 17.4	9	13	0.60	0.534	0.071
Fumarate	C00122	27.2 ± 6.0	53.5 ± 27.8	9	9	2.0	0.381	-0.156
3-Hydroxypropionate	C01013	143.3 ± 37.5	63.8 ± 21.1	7	6	0.45	0.072	0.399
Noradrenaline	C00547	2.0 ± 0.5	0.9 ± 0.5	7	3	0.46	0.151	0.304
Cadaverine	C01672	2.2 ± 0.7	23.7 ± 23.7	7	1	10.62	0.394	-0.147
Pelargonate	C01601	24.4 ± 7.4	24.0 ± 0.8	6	13	0.98	0.951	-0.044
Betaine aldehyde	C00576	20.7 ± 6.4	32.8 ± 5.5	6	10	1.6	0.168	-0.237
Histamine	C00388	1.3 ± 0.5	0.6 ± 0.3	6	4	0.49	0.269	0.282
Acetylcholine	C01996	0.5 ± 0.2	0.2 ± 0.1	5	3	0.37	0.145	0.373

Concentration data represent mean ± SEM of 12 and 13 samples of control and AIMD mice, respectively.
p values were calculated by Student's *t* test.
r values represent Pearson's correlation coefficient between concentration and PC1 factor value of PCA analysis.
